# Proteomics research on muscle-invasive bladder transitional cell carcinoma

**DOI:** 10.1186/1475-2867-11-17

**Published:** 2011-06-07

**Authors:** Hai Tao Niu, Zhen Dong, Gang Jiang, Ting Xu, Yan Qun Liu, Yan Wei Cao, Jun Zhao, Xin Sheng Wang

**Affiliations:** 1 Department of Urology, The Affiliated Hospital of Medical College Qingdao University, Qingdao, China; 2 Department of Radiology, The Affiliated Hospital of Medical College Qingdao University, Qingdao, China; 3 Department of Geratology, The 401th Hospital of PLA, Qingdao, China; 4 Department of Hematology, Qingdao University Medical College, Qingdao, China

## Abstract

**Background:**

Aimed to facilitate candidate biomarkers selection and improve network-based multi-target therapy, we perform comparative proteomics research on muscle-invasive bladder transitional cell carcinoma. Laser capture microdissection was used to harvest purified muscle-invasive bladder cancer cells and normal urothelial cells from 4 paired samples. Two-dimensional liquid chromatography tandem mass spectrometry was used to identify the proteome expression profile. The differential proteins were further analyzed using bioinformatics tools and compared with the published literature.

**Results:**

A total of 885/890 proteins commonly appeared in 4 paired samples. 295/337 of the 488/493 proteins that specific expressed in tumor/normal cells own gene ontology (GO) cellular component annotation. Compared with the entire list of the international protein index (IPI), there are 42/45 GO terms exhibited as enriched and 9/5 exhibited as depleted, respectively. Several pathways exhibit significantly changes between cancer and normal cells, mainly including spliceosome, endocytosis, oxidative phosphorylation, etc. Finally, descriptive statistics show that the P*I *Distribution of candidate biomarkers have certain regularity.

**Conclusions:**

The present study identified the proteome expression profile of muscle-invasive bladder cancer cells and normal urothelial cells, providing information for subcellular pattern research of cancer and offer candidate proteins for biomarker panel and network-based multi-target therapy.

## Background

Despite elaborate characterization of the risk factors, muscle-invasive bladder transitional cell carcinoma (BTCC) is still a major epidemiological problem whose incidence continues to rise each year [1]. The standard therapeutic methods of muscle-invasive BTCC are radical cystectomy (RC) followed postoperative care. Though there are much progress in surgical techniques and perioperative chemoradiation, the 5-year disease specific survival after RC remains 50-60% [2]. At present, the detailed mechanism for the carcinogenesis and development of invasive bladder carcinoma remains to be elucidated.

Though there were several proteomics research on muscle-invasive BTCC and make certain progress, the achievement of these researches were confined by the limited proteins identified from cancer tissue [3-5]. Nowadays, sophisticated proteomic, computational, and statistical tools offer us increased possibility of assimilating existing data to discover cancer biology and develop effective biomarkers for diagnosis and targeted therapy [6]. Meanwhile, with these technologies, some new concepts such as biomarker panel, subcellular proteomics study, and network-based multi-target therapy has been widely accepted [7-9].

Out of the above background, we perform shotgun strategy namely two dimensional liquid chromatography in conjunction with tandem mass spectrometry (2D-LC-MS/MS) for the direct analysis of complex mixtures as the initial step of our proteomics approach to understanding biology of invasive bladder cancer and to discover biomarkers. In order to exclude the interference of stromal elements and adjoining cells, purified cancer cells were obtained by laser capture microdissection. Based on the expression profile of purified cancer cells, gene ontology (GO) cellular component analysis was performed and the global feature of the expression profile as well as biomarker panel discovery was discussed. Aimed to achieve a systematical description of pathway changes and facilitate the network-based target therapy, Kyoto Encyclopedia of Genes and Genomes (KEGG) pathway database was retrieved.

## Methods

### Patients and tissue samples

A total of 4 paired muscle-invasive BTCC and normal urothelium samples (confirmed by two individual pathological diagnoses) were obtained from patients treated at the The Affiliated Hospital of Medical College Qingdao University immediately after radical cystectomy due to primary invasive bladder cancer. No patient had distant metastatic disease at cystectomy and no patient presented with carcinoma in situ. Pathologic staging and grading were performed according to the 2002 TNM classification system and World Health Organization criteria, respectively. The tumor and the adjacent microscopically normal urothelium (away from 5 cm) samples were rinsed in sterile PBS and snap frozen in liquid nitrogen within 30 min of removal. Table 1 lists the major clinical and pathological features of the 4 patients. The research protocol was approved by the Institutional Review Board and informed consent was obtained from patients.

**Table 1:** The major clinical and pathological features of the clinical samples

Patient	Age	Gender	Stage	Grade	Multifocality	Configuration
No.1	56	male	T2	G3	yes	Solid
No.2	48	male	T2	G3	yes	Solid
No.3	52	male	T2	G2	yes	Solid
No.4	61	female	T2	G3	yes	Solid

### Laser capture microdissection

**Figure 1:**
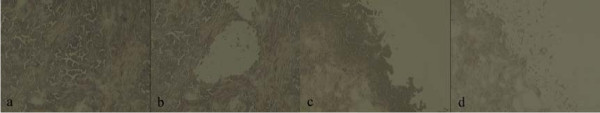
**Harvest the tumor cancer cells by LCM**. (A) Before LCM; (B) after LCM; Harvest the normal urothelium by LCM. (C) Before LCM; (D) after LCM

Eight-micrometer sections of freshly prepared tissues were stained with hematoxylin and eosin (H&E) to guide microdissection. The sections were air-dried and microdissected with a Leica AS LMD Laser Capture Microdissection System. Approximately 500, 000 shoots of tumor and normal urothelial cells from each specimen were microdissected and stored on microdissection caps at -80°C until lysed. Each cell population was determined to be 95% homogeneous by microscopic visualization of the captured cells. To avoid the degradation of protein, we capture the cells within 120 min each cap. Figure 1 shows a representative LCM of the sample. Laser capture microdissected cells were dissolved in lysis buffer (95 mM urea, 4% CHAPS, 40 mM Tris, 65 mM DTT). Samples were solubilized via sonication using 20 s bursts, followed by ice cooling (20 s), in a process that was repeated 5 times. After that, samples were centrifuged for 45 min at 15 000 rpm to remove DNA, RNA, and any particulate materials. The protein concentrations of samples were measured by Bio-Rad protein assay kit. All samples were stored at -80°C until use.

### Digestion of protein mixture for analysis

Samples prepared by LCM technology were deposited in precipitation solution (50% acetone/50% ethanol/0.1% acetic acid, sample volume: precipitation solution volume = 1:5) for at least 12 h at -20°C. The pellets were washed by 100% acetone and 70% ethanol, then redissolved in 6 mM guanidine-HCl/100 mM Tris (pH 8.3), and the concentrations were measured by Bio-Rad protein assay kit. Next, 250 μg of soluble proteins were reduced with DTT (final concentration 20 mM) and subsequently alkylated with IAA (final concentration 40 mM). After desalting and removal of hematoxylin and eosin by ultrafiltration with Microcon-10, the protein mixture was incubated with trypsin (trypsin: protein mixture = 1:30 w/w; Promega) at 37°C for 20 h.

### 2D-LC-MS/MS

All the two-dimensional high-performance LC separations were performed on a ProteomeX work station equipped with two capillary LC pumps. The flow rate of both salt and analytical pumps was at 120 μl/min and was about 1.5 μl/min after split. Nine different salt concentration ranges from 0, 25, 50, 75, 100, 150, 200, 400, and 800 mM ammonium chloride were used for step gradient. The mobile phases used for reverse phase were: A, 0.1% formic acid in water, pH 3.0; B, 0.1% formic acid in acetonitrile. Finnigan LTQ was used for peptide detection. The positive ion mode was employed and the spray voltage was set at 3.0 kV. The spray temperature was set at 170°C for peptides. Collision energy was set at 35%. After acquisition of full-scan mass spectra, ten MS/MS scans were acquired for the next ten most intense ions using dynamic exclusion. Peptides and proteins were identified using Sequest software, which uses the MS and MS/MS spectra of peptide ions to search against the publicly available IPI (International Protein Index) database. The protein identification criteria that we used were based on Delta CN (≥ 0.1) and Xcorr (one charge ≥ 1.9, two charges ≥ 2.2, three charges ≥ 3.75).

### GO and GO enrichment/depletion analysis

To take an overview of our proteomics analysis of invasive bladder cancer, we categorized the normal/cancer cells specific proteins as to GO assignments http://www.geneontology.org and GOfact software was used for the enrichment/depletion analysis [10]. For enrichment/depletion analysis, a test dataset (which is our identified proteins) and a reference set of GO annotation for the complete human proteome were in need. As per instructions on the GOfact webpage, the custom GO annotation for the reference set (of whole IPI human dataset) was created by extracting the GO annotations available for Human IPI IDs from EBI GOA Human 56.0 release. The analysis was done using fisher's exact test; the GO terms with *P *< 0.05 or *P *< 0.01 were selected as enriched/depleted or significantly enriched/depleted.

### Pathway analysis

ArrayTrack software was used for pathway analysis. ArrayTrack offers a simple query interface to retrieve information about human protein expression profile, and provides direct connections to related biological pathways available from Kyoto Encyclopedia of Genes and Genomes (KEGG). As to the ArrayTrack manual, the IPI names of the differentially expressed proteins were converted to SWISS-PROT names by ID convert tool prior to pathway analysis and then go to Pathway Search panel [11]. For statistical analysis, a *P *value for pathway enrichment was obtained using the fisher's exact test and *P *< 0.05 was considered statistically significant.

### Descriptive statistics on candidate biomarkers

The proteins that located in KEGG pathways by ArrayTrack were defined as potential biomarkers and the basic physico-chemical property of these proteins were further discussed. Skewness and kurtosis were used to test the distribution of the data. Mean values ± standard deviation was used to describe the central tendency and spread of normal distribution data. Interquartile-range (IQR) was used to give the basic characteristic of the variability skewness distribution data. All analysis was performed with SPSS^®^, version 16.0 and *P *< 0.05 was considered statistically significant.

## Results

### Identification of the proteins

From the 4 paired specimens we identified 990/983; 976/1032; 996/1058; and 1036/1011 proteins, respectively. This study mainly aimed to obtain global description of the biological characteristic of invasive bladder cancer. Therefore, we only analyzed the 885/890 proteins that commonly appeared in 4 paired specimens. There were 981 differentially expressed proteins between tumor and normal tissue. Among them, 488 proteins specifically expressed in cancer cells and 493 specifically expressed in normal cells. The smallest and largest molecular weight (MW) values observed in differentially expressed proteins were 7.34 and 1153.21 kDa, and the proteins were distributed across a wide isoelectric point (P*I*) range (3.67-12.14).

### GO and GO enrichment/depletion analysis

**Figure 2:**
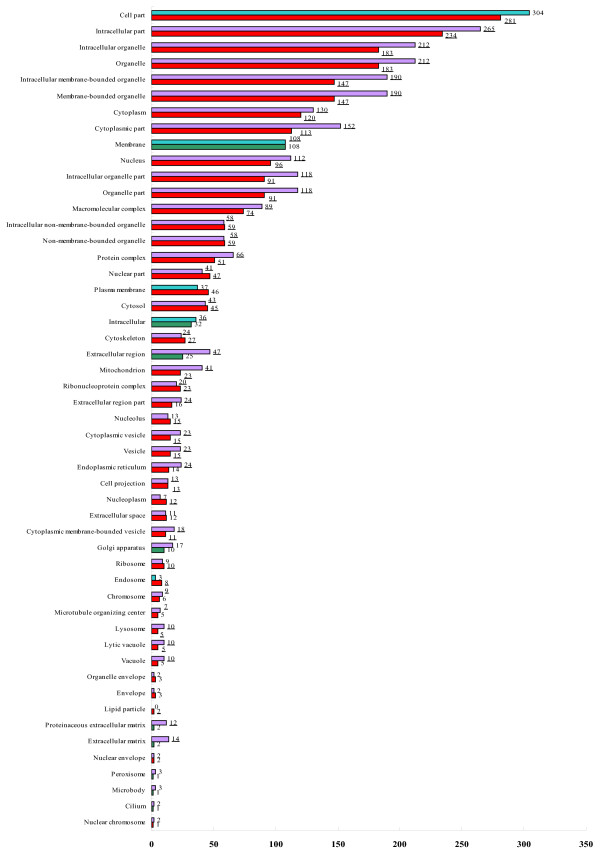
**Enriched/depleted GO cellular component terms for the set of tumor/normal cells specific proteins**. Purple/red indicates enriched terms in normal/cancer cells; light green/dark green indicate depleted terms in normal/cancer tissue. Underline indicates significantly enriched/depleted terms.

Of all the 488/493 proteins that were specifically expressed in tumor/normal cells, 295/337 with cellular component annotation. Compared with the entire list of the IPI (IPI Human, versions 3.13; 57050 protein sequences), there were 42/45 GO terms exhibited as enriched and 9/5 exhibited as depleted, respectively. Figure 2 shows enrichment/depletion analysis of cellular component. 27/24 proteins belong to the term of cytoskeleton, 23/40 belong to the term of mitochondrion, 11/24 proteins belong to the term of endoplasmic reticulum in tumor/normal tissue, respectively (Table 2).

**Table 2:** Proteins belong to cytoskeleton, mitochondrion, endoplasmic reticulum by GO enriched/depletion analysis

GO term	SWISS-PROT name	
	
	cancer	Normal
**Cytoskeleton**	RFA1, TMOD2, ACTH, DAG1, ACTZ, SRC8, FAK2, FSCN1, MYO3A, CTNA1, TBCA, TLN2, STOM, HINT1, NUCB1, SPR2E, DLG4, PTN14, ALDOC, LSP1, LIMA1, NHRF1, ABI1, CLIC4, CCDC6, NEBU, Q05331	ARAP3, JAK2, SSH1, MICA2, TPM3, MACF1, MYCB2, DPYL2, CK5P2, ABL2, STML2, FHOD3, SYNPO, COF2, NUDC, D9YZV3, TWF2, B4DQR8, UTRO, ANK2, GDIR2, TMOD3, LASP1, Q9Y427
**Mitochondrion**	GSHR, C1QBP, COX5B, ATPD, NDUS6, THIL, SSBP, CC90A, ATP5I, CH10, QCR7, QCR1, ADT2, ALDH2, IPYR2, PEDF, ACSS3, TIM8B, CLIC4, B2R608, ALDOC, Q5VTS5, GSHPx	B4DJA0, ETFA, IDHP, CATD, ECH1, PRDX4, DLDH, DHE3, COX2, HCD2, SODM, CH3L2, ECHM, PUR2, EFTU, PHB2, NDUA3, CY1, MCL1, S2533, AL1B1, ADHX, S10AA, C1TC, CY24B, SODC, CATB, QCR2, RPOM, STML2, ODO2, NDUA5, CYC, NEUL, HSP72, ATPO, PP1G, DECR, SYVC, AIFM1
**Endoplasmic reticulum**	RTN-1C, CAV1, EST1, EGFR, PDIA5, RCN3, RAB14, OST48, CNBP, ABI1, ACOC	HYOU1, SAR1B, FKBP2, SPTC1, PRAF3 HYEP, EXTL3, SC23A, HCD2, HNRPQ, LGAT1, THY1, TMED9, AN32A, LMAN1, ERD21, SERPH, DNJC1, CISD2, TXND5, APEX1, SC61B, SPCS3, CP19A

### Significant pathways and identification of potential biomarkers

After ID conversion, 745 of the 981 differential proteins with SWISS-PROT names and were analyzed by ArrayTrack. After analysis, 360 proteins were located at the well-known biological KEGG pathways and defined as potential biomarkers. The significantly altered pathways mainly include spliceosome, endocytosis, oxidative phosphorylation, etc. These pathways may take decisive role in the multistep tumorigenesis and development of invasive bladder cancer. Table 3 lists the major altered pathways.

**Table 3:** Major altered pathways

Pathway	SWISS-PROT name
**Spliceosome**	ACIN1, BCAS2, CRNKL1, EIF4A3, HNRNPA1, HNRNPA3, HNRNPM, HSPA2, LSM3, PRPF8, PUF60, SF3B1, SFRS13A, SFRS3, SFRS6, SFRS9, SNRPB, SNRPD2, SNRPD3, SNRPF, SNRPG, TRA2A, TRA2B
**Huntington's disease**	ATP5D, ATP5O, CLTA, CLTC, CLTCL1, COX2, COX5B, CYC1, CYCS, DLG4, DNAH2, EP300, NDUFA3, NDUFA5, NDUFS6, PLCB1, POLR2D, RCOR1, SLC25A5, SOD1, SOD2, UQCRB, UQCRC1, UQCRC2, VDAC2
**Alzheimer's disease**	ADAM10, ATP2A2, ATP2A3, ATP5D, ATP5O, CAPN2, COX2, COX5B, CYC1, CYCS, GRIN2C, HSD17B10, LRP1, NDUFA3, NDUFA5, NDUFS6, PLCB1, PPP3R1, TNF, UQCRB, UQCRC1, UQCRC2
**Endocytosis**	ARAP3, CLTA, CLTC, CLTCL1, EGFR, EHD1, EPN1, FAM125B, FLT1, HLA-A, HLA-B, HLA-C, HSPA2, MET, PDCD6IP, RAB11B, RAB11FIP3, RAB5C, VTA1
**Focal adhesion**	CAPN2, CAV1, COL4A2, CRK, DOCK1, EGFR, FLT1, FYN, ILK, ITGA5, ITGB1, MET, PIK3R3, PPP1CC, PPP1R12A, PRKCG, RAC2, THBS1, TLN2, TNXB
**Oxidative phosphorylation**	ATP5D, ATP5I, ATP5J2, ATP5O, COX2, COX5B, CYC1, NDUFA3, NDUFA5, NDUFS6, PPA2, TCIRG1, UQCRB, UQCRC1, UQCRC2
**Parkinson's disease**	ATP5D, ATP5O, COX2, COX5B, CYC1, CYCS, NDUFA3, NDUFA5, NDUFS6, SLC25A5, UQCRB, UQCRC1, UQCRC2, VDAC2
**Ribosome**	RPL10A, RPL21, RPL22, RPL31, RPL3, RPL5, RPL9, RPLP1, RPS12, RPS16, RPS19, RPS20, RPS5, RPS6, RPS7, RPS8
**Glycolysis/Gluconeogenesis**	ADH1A, ADH1B, AKR1A1, ALDH1B1, ALDH2, ALDOC, DLD, ENO2, GPI, PGAM2, PGM1, PKM2, TPI1
**Viral myocarditis**	ABL2, CAV1, CYCS, DAG1, FYN, HLA-A, HLA-B, HLA-C, HLA-DRA, HLA-DRB1, MYH15, RAC2
**Antigen processing and presentation**	CD74, CTSB, CTSS, HLA-A, HLA-B, HLA-C, HLA-DRA, HLA-DRB1, HSPA2, IFI30, PSME2
**Cardiac muscle contraction**	ATP1B3, ATP2A2, COX2, COX5B, CYC1, TPM2, TPM3, UQCRB, UQCRC1, UQCRC2

### Descriptive statistics on candidate biomarkers

**Figure 3:**
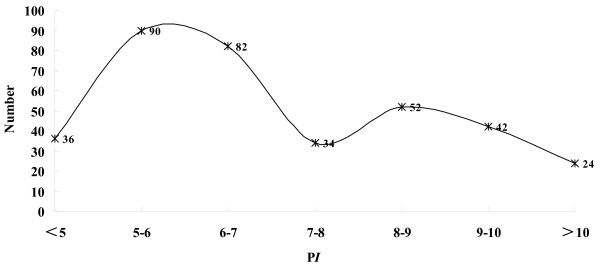
**Distribution of P*I *about the potential biomarkers**. Turning curve shows the global skewness distribution of the data. The data that P*I *≤ 8 (Skewness = 0.27 SE = 0.16; Kurtosis = 0.58, SE = 0.31, *P *> 0.05) and 8 < P*I *≤ 10 have normal distribution (Skewness = 0.27, SE = 0.25; Kurtosis = 0.61, SE = 0.49, *P *> 0.05).

**Figure 4:**
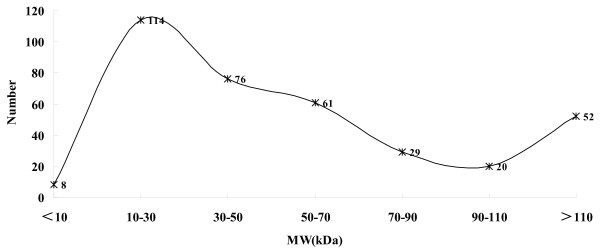
**Distribution of MW about the potential biomarkers**. Turning curve shows the global skewness distribution of the data. The data that MW ≤ 70 have skewness distribution (Skewness = 0.29, SE = 0.15; Kurtosis = 1.03, SE = 0.30, *P *> 0.05).

ArrayTrack defined a total of 360 proteins as potential biomarkers. Figure 3 and Figure 4 show the distribution of P*I *and MW about the potential biomarkers as well as the skewness distribution of the data. When the data is subdivided by P*I *equal 9 and MW equal 100 kDa, the IQR is 1.97 for P*I *≤ 9 and 1.14 for P*I *> 9, 33.28 for MW ≤ 100 and 130.84 for MW > 100, respectively. After further division and analyses, the data that P*I *≤ 8 and 8 < P*I *≤ 10 include 242, 94 proteins respectively have normal distribution and the mean ± SD is 5.98 ± 0.86 and 8.94 ± 0.51. Though the data that MW ≤ 70 looks like normal distribution, the pseudomorphism was see through by descriptive statistics with a P value > 0.05.

## Discussion

LCM technology can provide homogeneous sub-cell populations and minimising confounding variables, then we perform LCM prior to comparative proteomics analysis of muscle-invasive BTCC. After peptide mixture detection by 2D-LC-MS/MS, there were 885/890 proteins that commonly appeared in 4 paired tumor/normal specimens. Several biomarker candidates for cancer research in this profile have been previously reported. A critical functional consequence for observations connecting HNRNPA1 upregulation with cell proliferation, transformation has been demonstrated in a wide variety of cancers [12]. The activation of PLCB1 is an early and common response to stimulation of G protein-coupled receptors by these neuroendocrine growth factors and has been used for diagnostic and therapeutic target in lung cancer and multiple blood diseases [13]. Other examples are CYCS, COX5B, etc [14,15].

Cancer is now appreciated as a disease involving dysfunction of multiple subcellular pattern governing fundamental cell processes [16]. GO analysis on enlarged proteome coverage offer the predominance that select specific subcellular pattern to yield relatively simpler dataset for further research instead of whole-cell or whole-tissue proteome [17]. Compared with all human proteins deposited on IPI, the GO enrichment/depletion of the subcellular pattern mean the proteins in these categories are over/under presented in this profile and reflect the biologically specific categories of these data. By this way, shotgun strategy can understand carcinogenesis by subcellular level even without consideration of differentially abundant. As shown in Figure 2, our observation indicated that the global over/under represented terms in subcellular pattern of invasive bladder cancer and normal urothelium was quite consistent. Besides the global consistence, the proteins belonging to each pattern were completely different. The different proteins clustered in each identical pattern reflect different molecular networks can maintain the same basic subcellular function and behavior; meanwhile these differences may lead to carcinogenesis. Take the data listed in Table 2 for instant, the difference between the proteins that belong to same subcellular pattern offer further evidence that these subcellular patterns deserve extensively research in BTCC. Meanwhile, our outcome shows high degree of consistency with previous reports by that of substantial evidences indicate cytoskeleton, mitochondrion, and endoplasmic reticulum closely related with carcinogenesis [18-20].

The presentation of candidate proteins for biomarker panel discovery is additional contribution of enrichment/depletion analysis. Take the subcellular patterns of cytoskeleton, mitochondrion, endoplasmic reticulum that consist of the "hot-spots" in cancer research as examples. As shown in Figure 2 and Table 2, proteins in these three subcellular patterns were enriched in both cancer and normal cells. Though quantitative information cannot be obtained, specifically expressed proteins mean that either the proteins are expressed only in one tissue, or that significant difference exists in the expression of protein between the same sample amounts. As to the concept of GO cellular component enrichment/depletion analysis, the enriched GO terms indicate the active function of specific subcellular pattern and the proteins categorized in these terms were more likely to be present in biofluid, and more likely to be identified as biomarkers [21]. Based on the former description, we can primarily conclude that proteins categorized in these subcellular patterns might be effective candidates for the combination of independent, complementary markers. Most of them are first present in muscle-invasive BTCC research, such as caveolin-1 (CAV1), which shows tumor specifically expressed categorized to endoplasmic reticulum in our practice is a multifunctional scaffolding protein that regulates multiple cancer-associated processes related to malignant tumor progression [22]. Though most of the proteins listed in Table 2 have not been previous reported, the roles of these proteins in muscle-invasive BTCC biology deserve further investigation.

Proteomics pathway approach is necessary to understand multi-step carcinogenesis and facilitate network-based multi-target therapy [23,24]. The differentially expressed proteins mainly located at spliceosome, endocytosis, oxidative phosphorylation, etc. The spliceosome is a ribonucleoprotein complex involved in RNA splicing, that is, the removal of non-coding introns from precursor messenger RNA. Splicing events play an essential role in carcinogenesis and recent researches discover that the spliceosome is a target for novel compounds with anticancer activity [25]. Derailed endocytosis has emerged in the past 5 years as a multifaceted hallmark of malignant cells. Although it is unclear to what extent the endocytic and nuclear functions are interrelated, several of such proteins are implicated in the regulation of cell proliferation and carcinogenesis, arguing that their dual-function nature should be widely concerned. Pharmaceutical interception of the propensity of tumor cells to derail endocytic signaling and their adhesion receptors may constitute a novel target for cancer therapy [26]. Alterations in cellular bioenergetics contribute to the invasive, metastatic and adaptive properties typical in most tumors. Glycolytic inhibitors serve as a classical example of cancer metabolism targeting agents and several lines of evidence suggest that selective inhibition of tumor cell OXPHOS may be a rational approach for the treatment of cancers [27,28]. As previously described, by cellular pathway analysis, we found multiple pathway alterations between muscle-invasive BTCC and normal urothelium. The outcome of pathway analysis pointed out the direction of biotherapy. Furthermore, the proteins located in these pathways offer candidate biomarkers for design network-based multi-target therapy.

In this study, the largest molecular weight (MW) values observed in differentially expressed proteins was 1153.21 kea, and the proteins were distributed across a wide isoelectric point (P*I*) range of 3.67 to 12.14. After analysis by ArrayTrack, 65 proteins that P*I *> 9 and 63 proteins that MW > 100 kDa were defined as potential biomarkers. Based on these data, shotgun proteomics not only extenses the coverage of expressed proteome but also extenses the coverage of the expressed biomarkers in muscle-invasive bladder cancer. After categorizing the data by the detection range of routine 2-DE, the IQR is 1.97 for P*I *≤ 9 and 1.14 for P*I *> 9, 33.28 for MW ≤ 100 and 130.84 for MW > 100, respectively. Further analyses showed that P*I *≤ 8 and 8 < P*I *≤ 10 have normal distribution and the mean ± SD was 5.98 ± 0.86 and 8.94 ± 0.51, respectively. The aim of this procedure was for the guidance of selective gel excision in 2-DE technology. Though the overall distribution of P*I *and MW showed a skewed distribution, the P*I *distribution within a specific range shows certain regularity. As to the definition of normal distribution, all values within 1 SD encompass 68% of the data. Then, in the first dimension of 2-DE technology, 68% of the potential biomarkers centered in a range of 5.12 to 6.84 and 8.43 to 9.45 for that of P*I *≤ 8 and 8 < P*I *≤ 10, respectively.

## Conclusions

The present study identified 981 differential proteins between muscle-invasive BTCC and normal urothelium by shotgun strategy. GO enrichment/depletion analysis offer a number of candidate proteins to the combination of independent, complementary biomarkers and contribute to selection of specific subcellular pattern for further study. Pathway analysis reveals the differentially expressed proteins mainly located at spliceosome, endocytosis, oxidative phosphorylation, etc. Finally, descriptive statistics find that 68% of the potential biomarkers centered in a range of 5.12 to 6.84 and 8.43 to 9.45 for that of P*I *≤ 8 and 8 < P*I *≤ 10, respectively. These findings add our understanding of the mechanisms of carcinogenesis and may improve our ability to discover effective biomarkers for muscle-invasive BTCC.

## List of abbreviations

BTCC: bladder transitional cell carcinoma; LCM: Laser capture microdissection; 2D-LC-MS/MS: two dimensional liquid chromatography tandem mass spectrometry; GO: gene ontology; IPI: international protein index; KEGG: Kyoto Encyclopedia of Genes and Genomes; IQR: Interquartile-range; HNRNPA1: heterogeneous nuclear ribonucleoprotein a 1; PLCB1: phospholipase C beta 1; CYCS: cytochrome c; COX5B: cytochrome c oxidase subunit 5b; OXPHOS: oxidative phosphorylation;

## Competing interests

The authors declare that they have no competing interests.

## Authors' contributions

NHT performed the LCM and 2D-LC-MS/MS, and wrote the manuscript. DZ performed part of the 2D-LC-MS/MS and made the draft of the manuscript as co-first author. JG carried out the GO analysis. XT and LYQ carried out the pathway analysis. CYW and ZJ carried out the descriptive statistics on candidate biomarkers. WXS, as the corresponding author, designed the protocol and revised the manuscript. All authors read and approved the final manuscript.
